# The value of interleukin 6 as a peripheral diagnostic marker in schizophrenia

**DOI:** 10.1186/s12888-016-0866-x

**Published:** 2016-05-20

**Authors:** Kayla A. Chase, Jackson J. Cone, Cherise Rosen, Rajiv P. Sharma

**Affiliations:** The Psychiatric Institute, University of Illinois at Chicago, 1601 W. Taylor St., Chicago, IL 60612 USA; Department of Psychiatry, The University of California, 9500 Gilman Drive, La Jolla, San Diego, CA 92093 USA; Department of Neurobiology, The University of Chicago, 5812 S. Ellis, Chicago, IL 60637 USA; Jesse Brown Veterans Affairs Medical Center, 820 South Damen Avenue (M/C 151), Chicago, IL 60612 USA

**Keywords:** Schizophrenia, Lymphocyte, Interleukin-6, Biomarker, PBMC

## Abstract

**Background:**

Associations between a pro-inflammatory state and schizophrenia have been one of the more enduring findings of psychiatry, with various lines of evidence suggesting a compelling role for IL-6 in the underlying pathogenesis of schizophrenia.

**Methods:**

In this study, we examined IL-6 mRNA levels by real-time RT-PCR from fresh extracted peripheral blood mononuclear cells (PBMC) in normal controls and participants with schizophrenia.

**Results:**

We found that peripheral PBMC IL-6 mRNA levels, in the absence of any other information, reliably discriminated between a diagnosis of schizophrenia and normal controls. Furthermore, in participants with schizophrenia, we also found elevated levels of IL-6 mRNA with earlier ages of illness onset and worse positive symptom presentation, as measured by the Positive and Negative Syndrome Scale.

**Conclusions:**

These findings provide important and continued support for a pathophysiological role of inflammation in patients with schizophrenia. Future utilization of peripheral IL-6 mRNA levels could be clinically useful during an initial diagnosis and help tailor individualized treatment plans for patients with schizophrenia.

## Background

Patients with schizophrenia have altered immune function [1]. This, coupled with studies indicating the impact of maternal illness on offspring development [2, 3], strongly suggests a link between the inflammasome and schizophrenia. As such, interleukin 6 (IL-6), which can function as both a pro- and anti-inflammatory cytokine- has been examined for its role in the development, course, symptomology, and applicability as a biomarker of schizophrenia.

Various lines of evidence suggest a compelling role for IL-6 in the underlying pathogenesis of schizophrenia. First and foremost, IL-6 and sIL-6R serum levels are significantly elevated in patients with schizophrenia [4, 5], with implications for symptom severity [6]. The single-nucleotide polymorphism (SNP) G/C at position -174 of the IL-6 gene may also result in increased susceptibility to schizophrenia, although this seems to be highly dependent on environmental factors [7]. This SNP has also been linked to overall symptomology in schizophrenia [8, 9]. Maternal immune activation models of neurodevelopmental insults have heralded insight into the role of IL-6 and schizophrenia. Maternally produced cytokines can both cross the placenta [10] and induce an acute-phase reaction in response to immune challenges [11]. A single maternal dose of IL-6 results in schizophrenic-like behavioral changes in rodent offspring [12]. Furthermore, inhibiting IL-6 action in response to an immune challenge can normalize these behaviors in adult offspring [12]. Thus, multiple lines of evidence link inflammatory factors, specifically IL-6, with mental illness and related symptomology.

In this study, we demonstrate that IL-6 mRNA levels from freshly extracted peripheral blood mononuclear cells (PBMC) could be a useful and easily clinically accessible biomarker for a diagnosis of schizophrenia. There is a general lack of correlation between cytokine serum levels and PBMC gene expression [13]. As such, measurement of mRNA in PBMC is tissue specific and avoids interpretation regarding the source of serum levels of cytokines, which could emanate from the liver, muscle, adipose and other tissues. Additionally, we attempted to elucidate the role of IL-6 in disease onset and symptom presentation in schizophrenia.

## Methods

### Participants

The sample included 106 participants, including 53 patients with schizophrenia and 53 healthy individuals recruited from the University of Illinois at Chicago (UIC) medical center and surrounding community. All study procedures were approved by The Office for the Protection of Research Subjects at UIC. All participants provided written informed consent before participating in any study procedures. Patients with schizophrenia were assessed by experienced diagnosticians (MD or PhD) via Structured Clinical Interview for DSM Disorders (SCID) interview, DSM-IV-TR criteria and all available information. Clinicians established a consensus before final assignment to diagnostic group. All participants were between 21-65 years of age and in good physical health (with no reported infections or autoimmune diseases), and exclusion criteria were: a history of neurological disease or head trauma, lifetime history of substance/alcohol dependence or recent substance abuse, and pregnancy for women. Exclusionary criteria for control participants also included a major Axis I disorder (assessed by a SCID interview) and a known first-degree familial history of psychosis. Participant demographics are listed on Table 1.

**Table 1:** Means and standard deviations of participant demographics comparing healthy controls and participants with schizophrenia in PBMC samples

Demographic	Healthy Controls	Patients with Schizophrenia
Sex (M/F)	24/29	34/19
	t = 1.97; *p* = 0.052	
Age (Mean ± SD)	35.5 ± 11.4	36.8 ± 11.8
	t = -0.57; *p* = 0.57	
Race (%)		
Caucasian, non-Hispanic	17 (32)	4 (8)
Black, non-Hispanic	18 (34)	39 (73)
Asian or other Pacific Islander	11 (21)	3 (6)
Hispanic	7 (13)	7 (13)
	F(1,105) = 0.10; *p* = 0.75	
BMI (Mean ± SD)	29.3 ± 8.5	31.6 ± 7.7
	t = -1.36; *p* = 0.18	
Waist Circumference	39.8 ± 8.45	42.0 ± 8.17
	t = -1.26; *p* = 0.209	

Statistical differences between the various parameters were determined by a Student’s *t*-test or one way ANOVA, with results indicated

At the time of sampling, 55 % (*n* = 29) of the patients with schizophrenia were evaluated while hospitalized on the inpatient psychiatric unit and 45 % (*n* = 24) were evaluated in the psychiatric outpatient clinic. Prescribed antipsychotic medication was coded on all participants as follows: Haloperidol = 2, Fluphenazine = 2, Perphenazine = 2, Risperidone = 19, Olanzapine = 3, Quetiapine = 7, Ziprasidone = 3, Aripiprazole = 4, Lurasidone = 3, Iloperidone = 1, Paliperidone = 2, (a total of *n* = 48). Five patients were unmedicated at the time of the blood draw. Due to this heterogeneity, all antipsychotic use was converted to Chlorpromazine (CPZ) units [14, 15].

### Clinical measures

From all patients with schizophrenia, age of symptom onset was collected. Additionally, the Positive and Negative Syndrome Scale (PANSS) was administered to all clinical participants. The PANSS is used to generate measures of positive, negative, and general psychopathology through using a 7-point scale, with ratings ranging from 1 (symptom is absent) to 7 (symptom is extreme) [16].

### Peripheral blood mononuclear cell extraction

A blood sample was obtained by sterile venipuncture into a 0.5 M EDTA anticoagulant filled tube. Peripheral blood mononuclear cells (PBMC) were extracted utilizing the Ficoll-Paque® method (GE Healthcare Life Sciences) [17]. Subsequent washing of the cream-colored interlayer was performed using the Hanks Balanced Salt Solution (HBSS – Gibco #14170-161) to remove any remaining platelets, plasma or other contaminants. PBMC samples were pelleted at 2,000RPM for ten minutes at 10 °C and frozen in TRIzol reagent -80 °C until mRNA extraction.

### mRNA extraction and quantitative real-time RT-PCR

Total RNA was isolated using TRIzol reagent (Life Technologies) and treated with DNase (Ambion/Life Technologies AM1906) after extraction. Only total RNA extracts with an OD260/OD280 ratio above 1.96, indicating relatively pure RNA, were processed for RT-PCR, the remainder undergoing re-extraction using the standard phenol-chloroform extraction protocol. Total RNA was used to prepare cDNA via the Applied Biosystems High Capacity cDNA Reverse Transcription Kit (#4368814). For detection and measurement of expression, Fermentas Maxima SYBR Green/ROX qPCR Master Mix (#K0222) was used. PCR mixtures were run on a Thermo Scientific PikoReal real-time PCR System using the following conditions: 95 °C for 10 min, followed by 40 cycles of 95 °C for 30 s, 60 °C for one minute and 72 °C for one minute. Cycle threshold (CT) value was used for relative quantification, and all values were normalized to three housekeeping genes, GAPDH, TFRC and β-Actin (chosen for their previously, unpublished determined stability across diagnostic groups) using a geometric mean, and run in triplicate [18–20]. Primer sequences are listed in Table 2.

**Table 2:** Real-time RT-PCR mRNA primers utilized for this study

Primer Name	Left Primer	Right Primer
mRNA Expression Primers		
IL-6	AAAGAGGCACTGGCAGAAAA	AGCTCTGGCTTGTTCCTCAC
GAPDH	CGAGATCCCTCCAAAATCAA	TTCACACCCATGACGAACAT
TFRC	CACCAACCGATCCAAAGTCT	AAAATCCGGTGTAGGCACAG
βActin	TGAAGGTAGTTTCGTGGATGC	TCCCTGGAGAAGAGCTACGA

### Statistical analysis

SPSS (version 15.0 for Windows) was used for all statistical analyses. Analysis of all mRNA data was conducted on CT levels of the gene of interest, normalized to the geometric mean of three control genes. Data is presented as mean values ± standard error. A probability level of *p* < 0.05 was the criterion to achieve statistical significance.

## Results

### Shapiro Wilk analysis for normality

Normality of IL-6 mRNA distributions in both diagnostic groups was assessed by Shapiro-Wilk W tests where a *p* < 0.05 rejects the null hypothesis that the distribution is normal. Results indicate that the distribution for both controls and participants with schizophrenia was not normal, *p* = 0.001 and *p* = 0.008 respectively. Thus, for all further data analysis we utilized non-parametric tests, including the Mann-Whitney U, Spearman correlations, two-sample Kolmogorov-Smirnov test and the Receiver Operating Characteristic.

### Comparison of IL-6 mRNA expression levels in PBMC

**Fig. 1:**
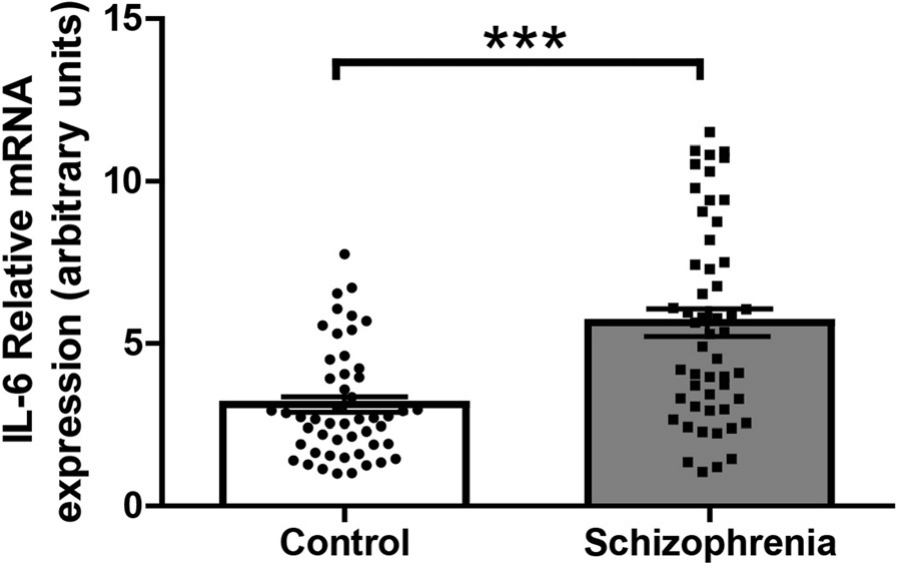
IL-6 mRNA levels in peripheral blood mononuclear cells (PBMC). IL-6 mRNA levels were significantly increased in participants with schizophrenia when compared to normal controls. Data presented as mean and SEM, *p* < 0.05

We performed a Mann-Whitney U analysis with PBMC IL-6 mRNA as the dependent variable and diagnosis as the independent variable. We discovered that participants with a diagnosis of schizophrenia had significantly higher IL-6 mRNA levels when compared to normal controls (*U* = 632.5, *p* < 0.001). There were no significant differences between diagnosis and measurements of sex, age, race, BMI or waist circumference, as determined by either a Student’s *t*-test or a One Way Analysis of Variance (ANOVA; Table 1). The mean value of IL-6 mRNA levels for control participants is 3.11 ± 1.7 (standard deviation). The mean value for PBMC IL-6 mRNA levels in participants with schizophrenia is 5.64 ± 3.0 (Fig. 1).

### IL-6 mRNA correlates with clinical scales

**Fig. 2:**
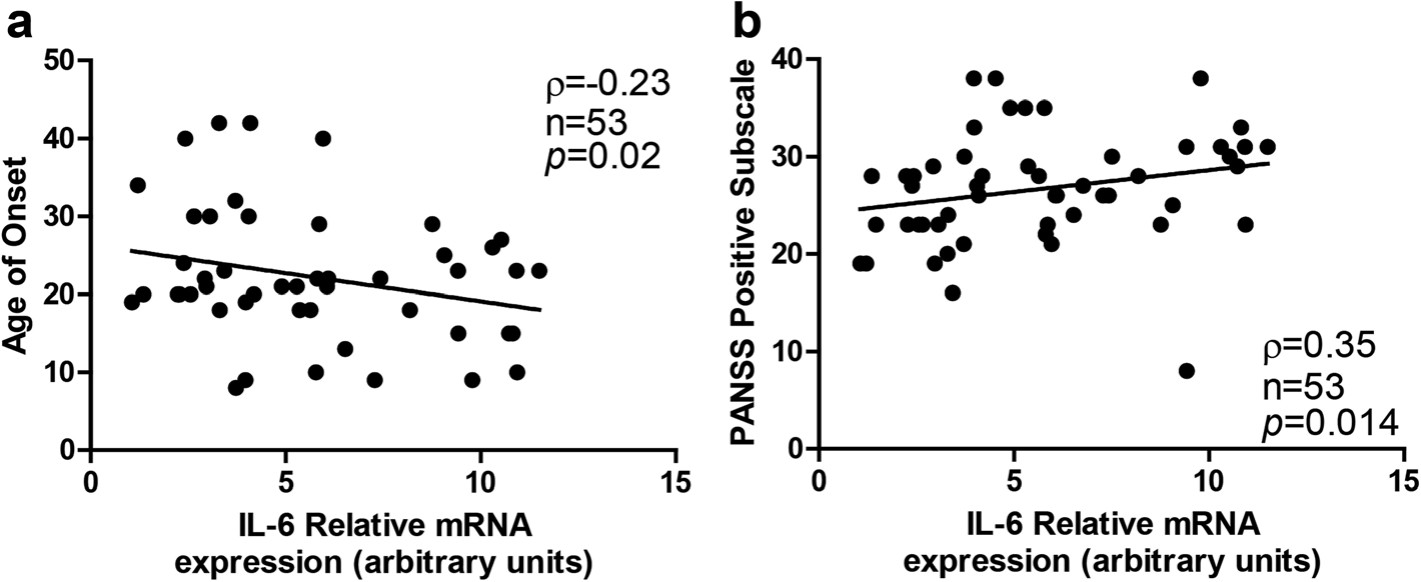
Clinical correlates with IL-6 mRNA levels (**a**) IL-6 mRNA levels were increased in participants with an earlier age of illness onset. **b** IL-6 mRNA levels were higher in participants with increased positive symptom presentation, as measured by the Positive and Negative Syndrome Scale (PANSS). Spearman rho values, sample size and significance levels are all listed on individual graphs

We found that higher PBMC IL-6 mRNA levels were associated with earlier onset of psychiatric symptoms (PBMC IL-6 mRNA x age of symptom onset; Spearman’s ρ = –0.23, *p* = 0.02; Fig. 2a). We also found that increases in IL-6 mRNA levels were correlated with increased presentation of positive symptoms, as measured by the PANSS (Spearman’s ρ = 0.35, *p* = 0.014; Fig. 2b).

### Two-sample Kolmogorov-Smirnov test

**Fig. 3:**
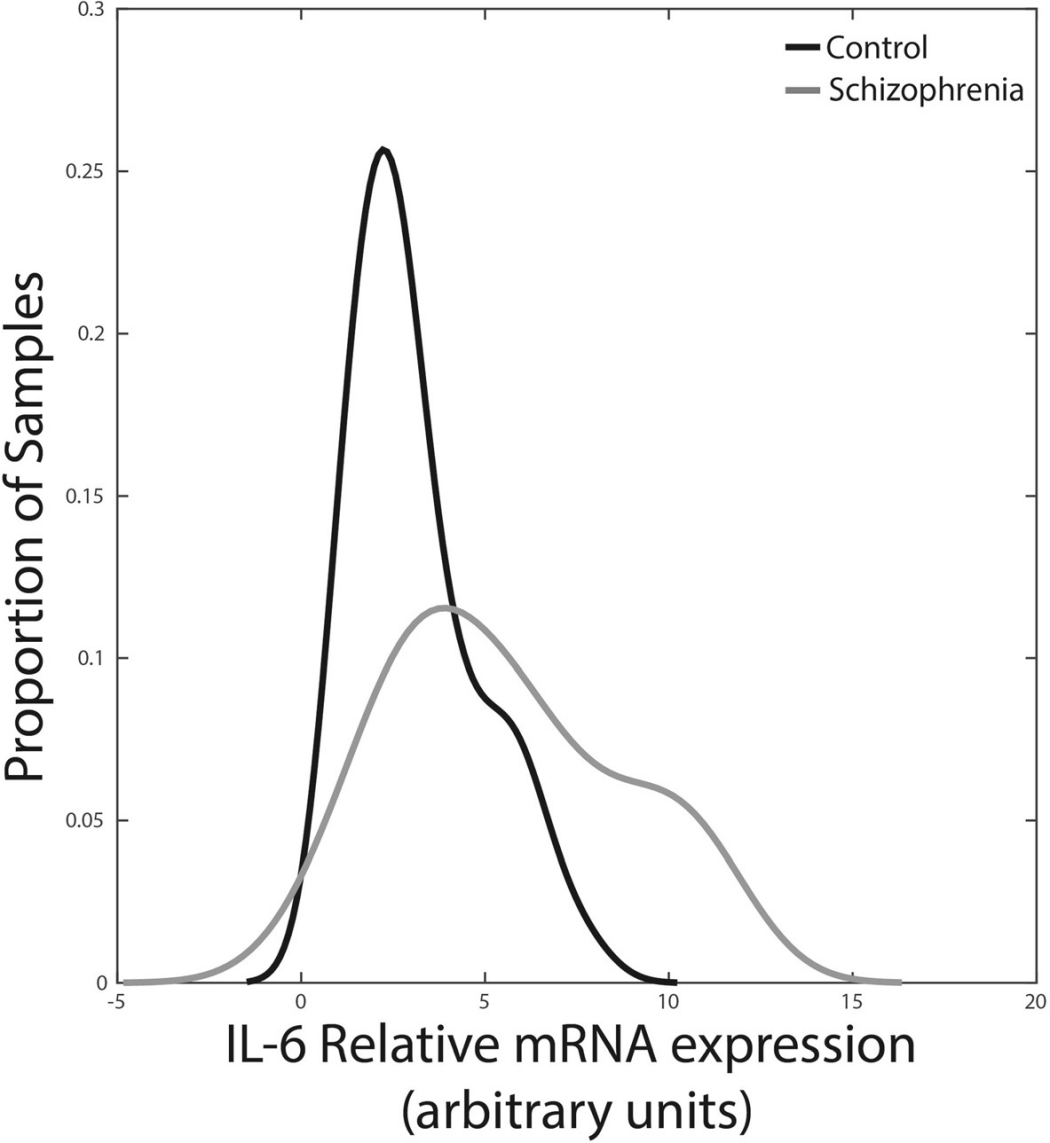
Distributions of IL-6 mRNA levels between controls and participants with schizophrenia. The two-sample Kolmogorov-Smirnov test is significant (*p* < 0.001), demonstrating that the two diagnostic groups originate from different distributions

A two-sample Kolmogorov-Smirnov (KS) test was used to compare the distributions of IL-6 mRNA levels between controls and participants with schizophrenia [21]. The KS test was significant (*p* < 0.001), supporting the hypothesis that the two samples originate from different distributions (Fig. 3).

### Receiver operating characteristic analysis

**Fig. 4:**
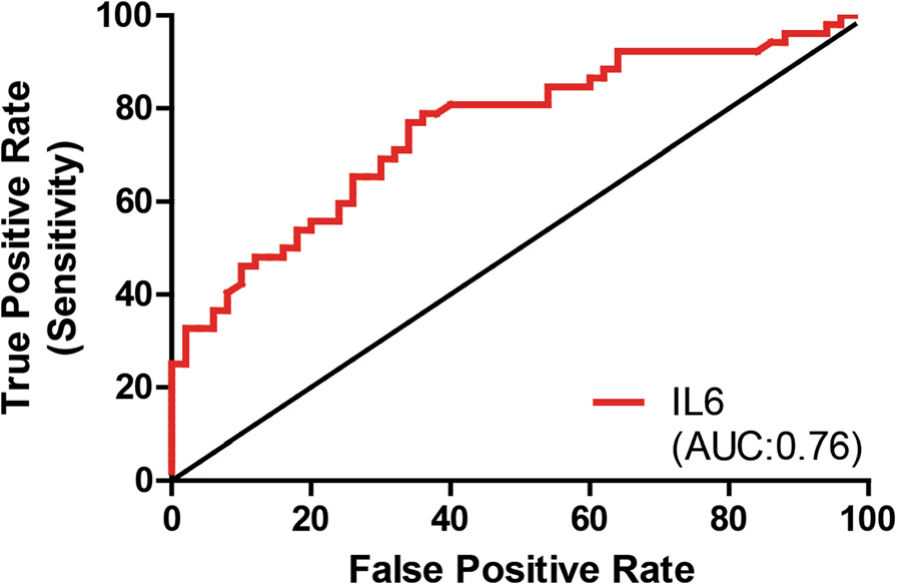
A receiver operating characteristics (ROC) curve analysis of the diagnostic value of IL-6 mRNA from primary blood mononuclear cells (PBMC). An area under the curve (AUC) value of 0.76 indicates the predictive capability of IL-6 mRNA levels for a diagnosis of schizophrenia in our sample

A receiver operating characteristics (ROC) curve was generated to examine the diagnostic value of using IL-6 mRNA as a binary classifier (patient with schizophrenia vs. controls). For this analysis, the area under the ROC curve (AUC) represents the predictive value of using only PBMC IL-6 mRNA levels to accurately differentiate between participants with schizophrenia and healthy controls across all of IL-6 mRNA levels found in our sample. While an AUC of 0.50 indicates there is no diagnostic value of IL-6 mRNA, an AUC greater than 0.50 indicates a greater than chance probability of successfully discriminating diagnoses from a randomly chosen sample of the IL-6 mRNA data. Notably, the AUC of IL-6 from PBMC was 0.76, with a 95 % confidence interval of 0.66-0.85 (*p* < 0.001; Fig. 4). This result demonstrates that IL-6 mRNA levels can reliably discriminate between a diagnosis of schizophrenia from normal controls in our sample (~25 % better than chance).

## Discussion

Our results demonstrate that IL-6 mRNA levels from freshly extracted PBMC are significantly upregulated in participants with schizophrenia when compared to healthy controls, thereby confirming our hypothesis. We also demonstrate that IL-6 mRNA levels are significantly elevated in patients who had an earlier age of onset, as well as those who demonstrated worse positive symptomatology. Finally, IL-6 levels may be a useful biomarker for a diagnosis of schizophrenia due to the predictive capabilities we demonstrate here.

Associations between a pro-inflammatory state and schizophrenia have been one of the more enduring findings of the field [4]. Our finding of an elevation in levels of IL-6 is consistent with the majority of the literature [6, 9, 22, 23]. One such study, by Frydecka, et al., [23] found that serum IL-6 levels were significantly higher in participants with schizophrenia when compared to healthy controls. Additionally, in their sample, elevated IL-6 serum levels were associated with longer duration of illness. Our study complements and extends these findings. Importantly, IL-6 levels examined in this manuscript are mRNA from peripheral blood mononuclear cells. IL-6 levels measured in the serum, as in the Frydecka paper, can originate from multiple tissues, including adipose, muscle and liver, with particularly confounding effects given the well known metabolic disturbances in the schizophrenia population [24, 25]. Thus the demonstrated elevated serum IL-6 levels could have originated at any number of locations, thereby making the true source, cause, and implications difficult to interpret. Additionally, the Frydecka et al., study found no associations between symptom presentation, as measured by the PANSS, and serum IL-6 levels, while we found a correlation with IL-6 mRNA levels and positive symptoms [23]. These differences may be attributable to the differences in type of biochemical sample examined between our two studies, paving the way for further discussion as to the importance of the location of the IL-6 signal and its role in mental illness.

Due to the heterogeneity of prescribed psychotropic medication in our naturalistic study, all antipsychotic use was converted to Chlorpromazine (CPZ) units. The impact of psychotropic medication on the inflammasome in participants with schizophrenia is a dynamic and novel field. Levels of proinflammatory cytokines are significantly elevated in female patients treated with clozapine, when compared to healthy controls [26]. However, these participants had significantly increased BMI levels when compared to normal controls, a phenotype associated with elevated cytokine levels, further complicating the interpretation. Additionally, treatment with olanzapine for 8 weeks reduces plasma IL-2 levels, but results in no changes in IL-6 or TNFa levels [27]. Treatment with chlorpromazine inhibits TNFa, but upregulates peripheral IL-10 levels in mice [28]. These findings all indicate that more research is needed regarding the uncontrollable variable of psychotropic medications play in the larger picture of inflammation and schizophrenia.

The association between IL-6 mRNA levels with age of onset and positive symptomatology is consistent with previous literature. In schizophrenia, serum IL-6 levels are positively correlated with PANSS Scores [29], an association our data replicates and extends on by examining this correlation with PBMCs. In schizophrenia, age of illness onset is a crucial determinant of clinical outcomes [30, 31], with a younger onset-age correlated with a worse disease prognosis and increased symptom presentation [32–34]. IL-6 levels are associated with younger psychosis onset, longer duration of illness and worse illness presentation [23], all indicators of worse disease course. IL-6 has been proposed to act as a “state marker” for schizophrenia, with elevated serum levels seen in first episode psychosis and acutely relapsed patients [35]. These listed studies are all in line with our findings that the underlying pathophysiology of schizophrenia is characterized by a low-grade inflammatory state. It is important to the field to present multiple lines of consistent data regarding elevated levels of IL-6 in participants with schizophrenia in independent cohorts. Additionally, further studies need to be conducted examining IL-6 mRNA levels in participants with schizophrenia when compared to other psychiatric disorders, specifically depression.

Previous work has explored the use of IL-6 as a clinical predictor, but for infection in febrile neutropenia [36], not schizophrenia. In schizophrenia research, the receiver operating characteristic analysis has been used to examine the predictive value of specific microRNAs not associated with the immune system [37, 38]. Our work merges these two concepts to examine the predictive value of PBMC IL-6 mRNA levels in schizophrenia. Interestingly, evidence indicates that IL-6 levels are increased in schizophrenia-risk subjects, with highest levels demonstrated in those who transitioned from risk to acute psychosis [39]. This suggests that IL-6 could serve as a pre-diagnostic tool for identifying individuals who are more likely to suffer from the disease. In the clinic, an especially useful aspect of the ROC is criterion; a cutoff that attempts to maximize the likelihood of a true positive (accurate diagnosis) while minimizing the chance of an incorrect diagnosis (false positive). In our data set, using a cutoff IL-6 mRNA value of 4.9 (arbitrary units) would result in a true positive rate of 53 %, while a false positive would occur 17 % of the time. If the cutoff value is increased, the chance of a true positive increases, while false positives decrease. For example, a cutoff of 6.0 in our sample would be five times more likely to make a correct diagnosis of schizophrenia based solely on IL-6 mRNA levels in PBMC. In the clinic, physicians would have access to much more patient data, and thus could adjust their criterion as needed to ensure an accurate diagnoses.

## Conclusions

Accessible biological markers are desperately needed to improve the timing and accuracy of a diagnosis of schizophrenia. These findings provide important support for a pathophysiological role for inflammation in patients with schizophrenia. While not strong enough to stand as a predictor of schizophrenia on its own, IL-6 mRNA levels may be yet another tool for clinicians to use during either an initial diagnosis or for tailoring individualized treatment plans for patients with schizophrenia. The application of accurate in vitro diagnostic tools that can accurately classify psychiatric disorders or identify disease subtypes could help to reduce illness duration and cost, including hospital services and loss of work, and improve treatment outcomes. Although such personalized medicine is still expensive, with the lightening speed of new technological development, these costs are rapidly decreasing [40]. In conclusion, although further studies using a larger cohort of participants are needed, data from the present study suggest that peripheral blood mononuclear cell level of IL-6 mRNA has diagnostic relevancy and predictive value in schizophrenia.

## Abbreviations

PBMC: peripheral blood mononuclear cells
PANSS: Positive and Negative Syndrome Scale
AUC: area under the curve
ROC: receiver operating characteristics
IL-6: interleukin 6
SNP: single-nucleotide polymorphism
SCID: Structured Clinical Interview for DSM Disorders
HBSS: Hanks Balanced Salt Solution
CT: Cycle threshold
CPZ: Chlorpromazine
KS: two-sample Kolmogorov-Smirnov test
